# An Unusual Presentation of Serotonin Syndrome and Subsequent Catatonia in a Patient With a Family History of Huntington’s Disease

**DOI:** 10.7759/cureus.45356

**Published:** 2023-09-16

**Authors:** Adela G Buciuc, Paula Traugott, Carlos R Danger

**Affiliations:** 1 Psychiatry and Behavioral Sciences, University of Miami Miller School of Medicine/Jackson Health System, Miami, USA; 2 Medicine, University of Buenos Aires, Buenos Aires, ARG; 3 Psychiatry and Behavioral Sciences, Jackson Behavioral Health Hospital, Miami, USA

**Keywords:** huntington disease, psychosis, neuroleptic malignant syndrome, serotonin toxicity, malignant catatonia

## Abstract

Neuroleptic malignant syndrome (NMS) and serotonin syndrome (SS) represent serious life-threatening conditions that share phenotypic and pathophysiologic features due to intricate interactions between the dopaminergic and serotoninergic systems. Malignant catatonia’s underlying pathophysiological mechanisms are poorly understood, but it is clinically difficult to distinguish it from NMS. Huntington’s disease (HD) is an autosomal dominant neurodegenerative disorder characterized by CAG expansion in exon 1 of the huntingtin (HTT) gene. Even though involuntary movements and lack of coordination are pivotal in HD, psychiatric manifestations are an integral part of it and may precede the emergence of chorea by years. The overlap in symptoms is noticeable for SS and NMS and distinguishing between the two may be challenging if exposure to both dopamine antagonists and serotoninergic agents exists. We present the case of a 48-year-old woman with an unusual presentation of serotonin syndrome and subsequent catatonia possibly overlapping with a neurodegenerative disorder, HD. This case report offers an interesting interconnection between three different syndromes that have tight pathophysiological and phenotypical associations.

## Introduction

Neuroleptic malignant syndrome (NMS), serotonin syndrome (SS), and malignant catatonia represent serious life-threatening conditions that share phenotypic and pathophysiologic features. Impairment of dopaminergic signalling is fundamental for developing NMS (whether through dopamine receptor antagonism or abrupt discontinuation of dopaminergic Parkinsonian agents), whereas for SS the hallmark is serotoninergic agonism [1]. Both syndromes represent the ends of a common pathophysiological framework due to the intricate interactions between the dopaminergic and serotoninergic systems. Catatonia is a clinical syndrome that occurs not only in patients with psychiatric disorders, but also in those with neurologic diseases and other medical conditions; its precise underlying pathophysiologic mechanisms are poorly understood, but the main hypothesis emphasizes the GABAergic and glutaminergic pathways [2]. Huntington’s disease (HD) is an autosomal dominant neurodegenerative disorder characterized by CAG expansion in exon 1 of the huntingtin (HTT) gene on chromosome 4p [3]. Even though involuntary movements and lack of coordination are pivotal in HD, psychiatric manifestations are an integral part of it and may precede the emergence of chorea by years

## Case presentation

A 48-year-old female presented to the emergency department of Jackson Behavioral Health Hospital (JBHH) referred from an outpatient community clinic for new-onset generalized stiffness. On examination, the patient presented with altered mental status, mutism, generalized tremors, distractable rigidity, and posturing. Other pertinent findings on physical examination: the patient was diaphoretic, with generalized hyperreflexia and clonus, tachycardic (117 beats/minute), and otherwise stable vital signs.

The patient recently immigrated to the United States; thus, no official medical records were available from her home country. Information was obtained from the family. Her past psychiatric history is significant for depression and schizophrenia and an unremarkable past medical history. The patient’s brother and sister were diagnosed with Huntington’s disease, but she never received a formal workup for it. She was recently admitted for acute exacerbation of paranoid schizophrenia in the context of medication non-adherence to a psychiatric inpatient unit and was discharged 6 days prior to JBHH presentation on 10 mg haloperidol, 30 mg duloxetine and 15 mg mirtazapine daily. Per relatives, the patient was at baseline upon discharge, but shortly after she became mute and refused alimentation (resulting in significant weight loss - approximately 4.5 kg).

**Figure 1:**
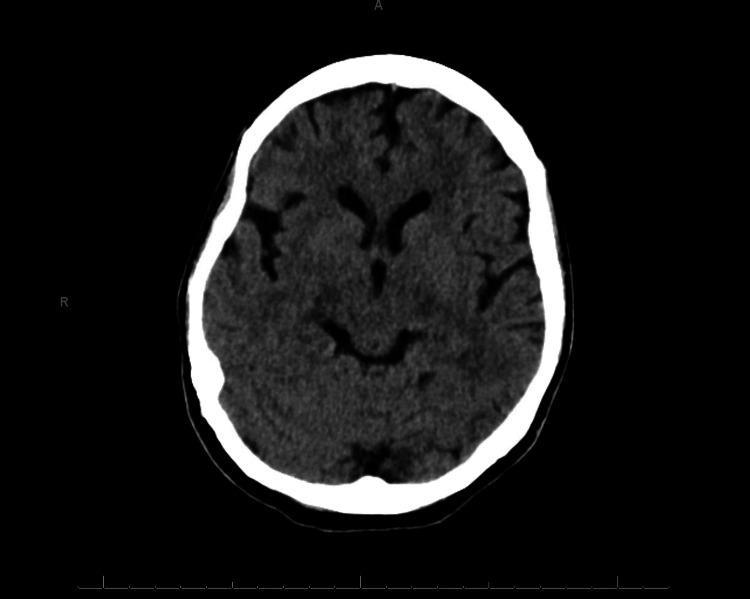
Non-contrast cerebral computer tomography. Axial plan. No evident caudate atrophy. No acute findings. Fronto-temporal atrophy present.

The patient was transferred to the medical emergency department for fluid resuscitation and further evaluation. Initial laboratory results (upon arrival at the emergency department) showed increased levels of lactic acid (7.6 mmol/L) and iron deficiency anemia. The complete blood count and creatine phosphokinase (CPK) levels were within normal limits. The urinary toxicology screen (drugs of abuse, salicylates, and acetaminophen) was negative. Our patient remained tachycardic (121 beats/minute), and mildly hypertensive (141/98 mmHg) with no hyperthermia. Fluid resuscitation, enoxaparin, and lorazepam IV were initiated. Upon neurology consult, the patient’s clinical presentation improved, and no rigidity was noted. She was lethargic but followed commands. Hyperreflexia with four beats of clonus bilaterally persisted. Non-contrast head computer tomography with no acute findings, and no clear caudate atrophy (see Figure 1).

On psychiatric revaluation, the patient was initially cooperative, and engaged in the interview, with depressed affect, spontaneous speech present, thought process was linear, with no signs or symptoms of psychosis. On repeat examination (after 1 hour) she was mute, posturing with waxy flexibility, hypertensive, and tachycardic. The patient scored 19 on the Bush Francis Catatonia rating scale. The patient’s clinical state partially improved to the first two doses of lorazepam (2 mg in total since she arrived in the emergency department), thus, the lorazepam dose was increased to 3 mg daily. Over the next two days, the patient’s clinical condition returned to baseline with normal neurological examination. Recommendations upon discharge - risperidone treatment was initiated for psychosis (2 mg daily as she had a good response in the past), lorazepam regime to be continued 3 days after discharge (2 mg daily) to maintain catatonia remission, genetic testing for Huntington’s disease in the outpatient setting.

## Discussion

Serotonin syndrome (SS) is a clinical diagnosis and can be thought of as a triad of clinical features consisting of altered mental status, neuromuscular hyperactivity (myoclonus, hyperreflexia, tremors), and autonomic dysregulation [1]. The intensity of clinical findings is thought to be dose-dependent and reflect the degree of serotonergic activity, whereas in NMS the reaction is idiosyncratic. Four different criteria have been proposed for SS diagnosis (Sternbach, Hegerl, Radomski, and Hunter), with Hunter criteria being regarded as the gold standard [4]. The Hunter Serotonin Syndrome Criteria require exposure to a serotoninergic drug and one of the following features to make an SS diagnosis: spontaneous clonus; inducible clonus with agitation/diaphoresis; ocular clonus with agitation/diaphoresis; tremor and hyperreflexia; or hypertonia, temperature >100.4°F and ocular or inducible clonus (Table 1) [4].

**Table 1:** Hunter Criteria for Serotonin Syndrome Adapted from [4]

In the presence of a serotoninergic agent
If spontaneous clonus is present, then serotonin toxicity: YES
Else, if inducible clonus is present AND agitation OR diaphoresis, then serotonin toxicity: YES
Else, if ocular clonus is present AND agitation OR diaphoresis, then serotonin toxicity: YES
Else, if tremors are present AND hyperreflexia, then serotonin toxicity: YES
Else, if hypertonia is present AND temperature >100.4°F AND ocular clonus is present OR inducible clonus is present, then serotonin toxicity: YES

In contrast to SS, NMS is a diagnosis of exclusion. Patients with exposure to dopamine antagonists or abrupt discontinuation of dopaminergic Parkinsonian agents develop altered mental status, muscular rigidity, and autonomic instability [1], with the latter being the feature that helps to differentiate NMS from uncomplicated catatonia. According to the *Diagnostic and Statistical Manual of Mental Disorders 5* (DSM-5) [5], a diagnosis of NMS requires exposure to a dopamine-blocking agent, severe muscular rigidity, fever, and at least two of the minor criteria: diaphoresis, dysphagia, tremor, incontinence, altered level of consciousness, mutism, tachycardia, elevated or labile blood pressure, leucocytosis, and elevated CPK (Table 2). According to many diagnostic criteria, fever is a defining symptom of NMS, however, many atypical presentations of NMS [6] (especially in the early course) are constantly reported in the literature.

**Table 2:** Neuroleptic Malignant Syndrome criteria CK: creatine phosphokinase; Fe: ferrous Adapted from DSM-5 [5]

Exposure to dopamine antagonism within 72 hours prior to the beginning of symptom development
Hyperthermia (>100.4°F or >38.0°C on at least two occasions, measured orally)
Profuse diaphoresis
Generalized rigidity (“lead pipe”), associated with other neurological symptoms (e.g., tremor, sialorrhea, akinesia, dystonia, trismus, myoclonus, dysarthria, dysphagia, rhabdomyolysis)
Mental status: delirium or altered consciousness (ranging from stupor to coma)
Autonomic nervous system activation and instability: tachycardia (rate > 25% above baseline), blood pressure elevation (systolic or diastolic >25% above baseline) or fluctuation (>20 mmHg diastolic change or >25 mmHg systolic change within 24 hours), hypertonia (>25% above baseline or with fluctuation), sialorrhea, urinary incontinence, pallor, tachypnea (>50% above baseline), respiratory distress (can lead to sudden respiratory arrest)
Laboratory findings: ­leukocytes, ­CK (of at least four times the upper limit of normal), ­serum muscle enzymes, ­catecholamines, ¯Fe, metabolic acidosis, hypoxia
Neuroleptic malignant syndrome must be distinguished from other serious neurological or medical conditions as well as from similar syndromes resulting from the use of other substances or medications

The overlap in symptoms is noticeable for SS and NMS and distinguishing between the two may be challenging if exposure to both dopamine antagonists and serotoninergic agents is present. Although their presentations are similar, some of the features may direct the clinician towards a specific diagnosis. Onset and resolution of symptoms are more rapid in SS [7] (usually 24-48 hours after stopping the offending agent) than NMS (days to weeks). Severe muscular rigidity with hyporeflexia is more suggestive of NMS, whereas neuromuscular excitability (hyperreflexia, myoclonus) is the hallmark of serotonin toxicity [7]. Laboratory findings most consistent with NMS are leucocytosis, increases in CPK levels, and low serum iron levels [7]. Gastrointestinal symptoms (vomiting, diarrhea) are usually absent in NMS but present in SS.

The mainstay of treatment in mild and moderate cases, besides stopping the offending agent, is represented by supportive treatment (rehydration and electrolyte repletion; close monitoring of vital signs, and of cardiac, respiratory, and renal functions is required). Anticoagulation therapy should be initiated, as patients have a high risk of thromboembolism and disseminated intravascular coagulation. Aspiration prevention is another important aspect in this population of patients. In refractory cases, in NMS, dopamine agonists (bromocriptine, amantadine) are used to counteract the dopamine receptors blockade; dantrolene, which is a direct skeletal muscle relaxant is another agent that may be used to treat rigidity and fever. Failure to respond to these measures may require electroconvulsive therapy. In contrast, in SS refractory cases, cyproheptadine, an antihistaminic that is a 5-HT1A and 5-HT2A inhibitor as well, should be considered. Immediate sedation, paralysis, and tracheal intubation may be considered in patients with SS and a temperature >41.1 °C (106 °F) [7].

Our patient presented to the Psychiatry emergency department with altered mental status, mutism, distractable rigidity, hyperreflexia with bilateral myoclonus, and persistent tachycardia after six days of new treatment initiation of 10 mg haloperidol, 30 mg duloxetine, and 15 mg mirtazapine daily. The confounding factor for our patient’s clinical presentation was the family history of Huntington’s disease. Even though the motor symptoms of manifest HD are fairly characterized, the sequence of progression remains uncertain with much conflicting information. It is well known that in the manifest disease and its late stages, the chorea limits functioning, furthermore, dystonias and athetosis appear [8]. In the early stage of HD, presentation is variable; the first motor abnormalities that have been described in the literature to appear are brisk reflexes, diminished rapid alternating movements, abnormal extraocular movements, and mild chorea [8]. The presumptive diagnosis was SS versus NMS. Even though in NMS, hyperreflexia and myoclonus are rare, they can be a part of the clinical presentation of a patient who is in the early phase of HD with superimposed NMS. Upon initial laboratory results, the leukocyte count was within normal limits and CPK levels were normal, findings which are not suggestive of NMS. Atypical presentations of NMS with normal levels of CPK have been described in the literature [6,9]; furthermore, in the initial stages of the syndrome when rigidity is not well developed, CPK levels may be within normal limits. The patient’s clinical status improved under supportive care and benzodiazepine therapy. After 24 hours from the initial presentation, the signs of neuromuscular hyperactivity diminished.

In addition to motor abnormalities, cognitive and behavioral changes are part of HD. There are many contrasting findings regarding the sequence of progression of the three aspects mentioned. Psychiatric manifestations in manifest HD have a high prevalence and resemble psychiatric disorders (depression, mania, OCD, primary psychotic disorders). It has been said that chorea may be preceded by major depression disorder and other personality changes (irritability, restricted affect) by years [10]. Poorly systematized paranoia resembling schizophrenia is one of the most common manifestations of psychotic disorders in early HD and is associated with flattened affect, irritability, and poor impulse control.

One of the limiting factors of our case report is that information regarding our patient’s past medical history was obtained from her family, with no access to official medical records from her home country (“patient has been depressed for many years” before her first psychotic episode). The patient will follow up for genetic testing for HD in the outpatient setting.

## Conclusions

This case report offers an interesting interconnection between three different syndromes that have tight pathophysiological and phenotypical associations. It is of the utmost importance to promptly recognize and differentiate SS from NMS, as treatment should be started without delay to avoid any life-threatening consequences. Furthermore, it is relevant to highlight that scientific literature regarding psychiatric manifestations in the initial stages of HD is insufficient, with conflicting information. The underlying pathophysiology mechanisms leading to psychotic manifestations and their clinical presentations are poorly understood, thus making these enticing avenues for research which may bring along new diagnostic and early treatment strategies to improve prognosis in this segment of the population.
